# TGFβ Blockade by Inhibition of Enolase-1-Mediated Plasmin Targets Tumor-Associated Macrophages in Pancreatic Ductal Adenocarcinoma

**DOI:** 10.32604/or.2026.077930

**Published:** 2026-07-16

**Authors:** Mao-Lin Chen, I-Che Chung, Kevin Chih-Yang Huang, K. S. Clifford Chao, Ta-Tung Yuan

**Affiliations:** 1 Department of Research and Development, HuniLife Biotechnology Inc., Taipei City, Taiwan; 2 Department of Biomedical Imaging and Radiological Science, China Medical University, Taichung, Taiwan; 3 Translation Research Core, China Medical University Hospital, Taichung, Taiwan; 4 Cancer Biology and Precision Therapeutics Center, China Medical University, Taichung, Taiwan; 5 Office of Research and Development, Asia University, Taichung, Taiwan; 6 Center of Proton Therapy, China Medical University Hospital, Taichung, Taiwan; 7 Graduate Institute of Biomedical Sciences, China Medical University, Taichung, Taiwan; 8 Department of Radiation Oncology, China Medical University Hospital, Taichung, Taiwan

**Keywords:** ENO1, TGFβ activation, pancreatic ductal adenocarcinoma, monoclonal antibody, tumor microenvironment, tumor-associated macrophage

## Abstract

**Background:** Pancreatic ductal adenocarcinoma (PDAC) is characterized by an immunosuppressive and metabolically rewired tumor microenvironment (TME). Although α-enolase (ENO1) is frequently overexpressed in PDAC and associated with poor prognosis, its functional role in TME remodeling remains unclear. This study investigated the role of ENO1 in plasmin-dependent transforming growth factor β (TGFβ) activation and metabolic adaptation in PDAC and evaluated the therapeutic potential of HuL001, a first-in-class humanized anti-ENO1 monoclonal antibody. **Methods:** ENO1 expression and clinical relevance were evaluated in PDAC tissues by immunohistochemistry. Mechanistic studies were performed using PDAC-monocyte co-culture systems, reverse transcription-quantitative PCR (RT-qPCR), enzyme-linked immunosorbent assay (ELISA), flow cytometry, and cell viability assays. The therapeutic effects of HuL001 were further assessed in multiple PDAC xenograft models. **Results:** Surface ENO1 expression was elevated in advanced PDAC and was associated with poorer overall survival. HuL001 suppressed monocyte-driven PDAC proliferation and reduced factors associated with M2-like macrophages (alternatively activated macrophages), including *TGFB1*, *IL10*, and *VEGFA*. Mechanistically, HuL001 inhibited ENO1-dependent plasmin-mediated activation of latent TGFβ and attenuated TGFβ-induced ENO1 expression, plasmin activity, hexokinase 2 (HK2) expression, and lactate production. *In vivo*, HuL001 inhibited PDAC growth and enhanced the antitumor efficacy of gemcitabine in three xenograft models, with greater suppression of tumor growth, intratumoral lactate, and active TGFβ than gemcitabine alone. **Conclusion:** These findings identify ENO1 as a functional driver of plasmin-dependent TGFβ activation and metabolic adaptation in PDAC and support HuL001 as a promising therapeutic strategy, particularly in combination with gemcitabine.

## 1 Introduction

Pancreatic ductal adenocarcinoma (PDAC) is one of the most aggressive and treatment-resistant solid malignancies, with a 5-year survival rate below 10% [1]. Most patients are diagnosed at a locally advanced or metastatic stage, and current cytotoxic therapies provide only limited benefit [2,3]. Beyond late diagnosis and intrinsic metastatic potential, PDAC progression is strongly shaped by a hostile tumor microenvironment (TME) that promotes immune evasion, metabolic adaptation, and resistance to therapy [4,5,6]. These challenges highlight the urgent unmet need for therapeutic strategies that target both tumor cells and the tumor-supportive microenvironment.

Within the TME, tumor-associated macrophages (TAMs) are critical drivers of disease progression [7]. In PDAC, TAMs predominantly exhibit an M2-like phenotype (alternatively activated macrophages), which is consistently associated with poor clinical outcomes [8,9]. M2-like TAMs exert tumor-supportive effects through the secretion of immunosuppressive and pro-angiogenic cytokines such as transforming growth factor-beta (TGFβ), interleukin-10 (IL-10), and vascular endothelial growth factor (VEGF) [10]. TAMs also interact with other stromal components, particularly cancer-associated fibroblasts (CAFs), to exacerbate hypoxia, desmoplasia, and immune suppression [11]. Despite their importance in PDAC, effective therapeutic strategies targeting TAM-driven tumor-supportive signaling remain limited [12].

Among these factors, TGFβ is a key regulator of the PDAC microenvironment that promotes immune suppression, fibrosis, and therapeutic resistance in advanced disease [13]. Although TGFβ acts as a tumor suppressor in early stages, it drives tumor progression at later stages by inducing epithelial-mesenchymal transition, activating CAFs, and promoting M2-like TAM polarization [14]. TGFβ is secreted as a latent complex that requires activation to initiate receptor-mediated signaling. Reported activation mechanisms include proteolytic cleavage by plasmin, matrix metalloproteinases, and thrombospondin-1; integrin-mediated mechanical force; and physicochemical stimuli such as acidic pH, heat, and reactive oxygen species [15]. Notably, TGFβ was among the stimuli reported to increase cell-surface enolase-1 (ENO1) mobilization in MDA-MB-231 breast cancer cells [16]. Together, these findings suggest a potential feed-forward loop between ENO1 and TGFβ activation. Because therapeutic targeting of TGFβ has shown limited success due to systemic toxicity and incomplete clinical benefit [17], targeting ENO1 may provide an alternative strategy to interfere with TGFβ-driven stromal and immune remodeling in PDAC.

ENO1 (or α-enolase) is a multifunctional protein best known as a glycolytic enzyme that catalyzes the conversion of 2-phosphoglycerate to phosphoenolpyruvate [18]. Under cellular stress, ENO1 translocates to the cell surface, where it acts as a plasminogen-binding receptor to facilitate local plasmin generation and extracellular matrix degradation [19]. These dual functions position ENO1 as a potential mediator of both tumor metabolism and stromal remodeling. In PDAC, ENO1 is frequently overexpressed and associated with poor clinical outcomes [20,21] and gemcitabine resistance [22,23,24], although its role in remodeling the TME remains unclear. Given its role in plasmin generation and the potential ENO1/TGFβ feed-forward relationship, ENO1 may represent an upstream modulator of latent TGFβ activation and a therapeutic target in PDAC. To investigate this hypothesis, we employed our proprietary ENO1-targeting antibody HuL001 to examine the ENO1/TGFβ pathway in cancer development using PDAC cells and animal models. HuL001 has completed a first-in-human clinical trial with favorable safety and pharmacokinetic profiles, supporting its further evaluation in ENO1-expressing malignancies. 

In the present study, we investigated the pathophysiological role of ENO1 in PDAC, with particular emphasis on its involvement in tumor microenvironment remodeling and metabolic adaptation. Specifically, we aimed to determine whether ENO1 contributes to PDAC progression through plasmin-dependent activation of TGFβ, TAM-related tumor-promoting signaling, and glycolytic reprogramming. We also evaluated the therapeutic potential of HuL001, a humanized anti-ENO1 monoclonal antibody, as monotherapy and in combination with gemcitabine in PDAC models. We hypothesized that targeting ENO1 with HuL001 would disrupt this tumor-supportive signaling axis and thereby attenuate PDAC progression.

## 2 Materials and Methods

### 2.1 Patient Samples and Tissue Collection

Formalin-fixed paraffin-embedded (FFPE) tissue samples from 53 patients diagnosed with resectable PDAC were obtained from [China Medical University Hospital, Taichung, Taiwan], following approval by the institutional review board (IRB No. CMUH111-REC2-111) and in accordance with the Declaration of Helsinki. The cohort included 32 male and 21 female patients, with a median age of 64.5 years (range 39–83). Paired tumor tissues and adjacent normal tissues were collected from patients undergoing surgical resection between [2007–2015]. Patients with surgical resection and follow-up in China Medical University Hospital were included in this study. Patients with complicated symptoms were excluded. The pathological TNM stage was determined by postoperative pathological examination according to the 7th edition of the AJCC staging system. For clinicopathological analysis, patients were stratified into pTNM1-2 and pTNM3-4 subgroups. Written informed consent was obtained from all participants.

### 2.2 Immunohistochemistry (IHC)

IHC staining was performed to assess ENO1 expression in PDAC tissues. FFPE tissue sections (4 μm) were deparaffinized in xylene, rehydrated through graded ethanol, and subjected to heat-induced antigen retrieval using citrate buffer (pH 6.0) in a microwave oven for 15 min. Endogenous peroxidase activity was blocked by incubating sections with 3% hydrogen peroxide for 10 min at room temperature. Tissue sections were then blocked with 5% normal goat serum (#S-1000-20, Vector Laboratories, Burlingame, CA, USA) for 30 min and incubated overnight at 4°C with an anti-ENO1 primary antibody (#ab227978, Abcam, Cambridge, USA, dilution 1:200). After washing, sections were incubated with horseradish peroxidase (HRP)-conjugated secondary antibody (#ab6721, Abcam, dilution 1:500) for 30 min at room temperature. Signal was developed using 3,3′-diaminobenzidine (DAB) substrate system (comprising DAB substrate #TA-060-QHSX and chromogen #TA-002-QHCX, Thermo Fisher Scientific, Waltham, MA, USA), and nuclei were counterstained with hematoxylin (Gill II, #3801522, Leica Biosystems, Richmond, IL, USA). Sections were dehydrated, cleared, and mounted with coverslips for microscopic analysis (OLYMPUS BX53, Tokyo, Japan).

### 2.3 Evaluation of ENO1 Expression and H-Score Analysis

Stained slides were examined using a BX50 light microscope (Olympus, Tokyo, Japan, ×200 magnification). Membranous ENO1 immunoreactivity was semi-quantitatively assessed using the H-score method. The H-score was calculated by multiplying the percentage of positively stained cells (0–100%) by staining intensity (0 = negative, 1 = weak, 2 = moderate, 3 = strong), resulting in a score ranging from 0 to 300. Two independent pathologists blinded to clinical data performed the scoring, and mean scores were used for analysis. The mean H-score was defined as a cut-off to stratify into high and low subgroups (Mean = 86.7).

### 2.4 Patient Stratification and Survival Analysis

Patients were stratified into high and low ENO1 expression groups based on the median H-score. Overall survival (OS) was defined as the time from initial diagnosis to death or last follow-up. Kaplan–Meier survival curves were generated, and statistical differences between groups were evaluated using the log-rank test in GraphPad Prism 7 statistical software (GraphPad Software, San Diego, CA, USA). Hazard ratios (HR) and 95% confidence intervals (CI) were calculated using Cox hazard model and univariate analysis to determine the risk associated with high ENO1 expression.

### 2.5 Cell Culture and Reagents

The human PDAC cell lines MIA PaCa-2 (BCRC 60139) and PANC-1 (BCRC 60284) were obtained from the Bioresource Collection and Research Center (BCRC, Hsinchu, Taiwan) and authenticated using short tandem repeat (STR) profiling at BCRC. PANC-1/M cells were a gift from Dr. Neng-Yao Shih as previously described [25]. The human monocytic cell lines THP-1 (BCRC 60430) and U937 (CRL-1593.2) were obtained from the BCRC and American Type Culture Collection (ATCC, Manassas, VA, USA), respectively, and were authenticated by STR profiling at the respective cell banks. All cells were cultured in complete Dulbecco’s Modified Eagle’s Medium (DMEM, #11995065, Gibco, Thermo Fisher Scientific), consisting with 10% fetal bovine serum (FBS) (#26140079, Gibco) and 50 U/mL penicillin-streptomycin (#15140122, Gibco). Cells were maintained at 37°C in a humidified atmosphere containing 5% CO_2_. According to the suppliers’ quality control documentation, the cell lines obtained from BCRC and ATCC were tested for mycoplasma contamination prior to distribution. Tranexamic acid (TXA; #T1810000, European Pharmacopoeia/EDQM, Strasbourg, France), recombinant human TGFβ1 protein (#100-21, PeproTech, Cranbury, NJ, USA), human latent TGFβ1 (#HY-P73426, MedChemExpress, Monmouth Junction, NJ, USA), and Matrigel (#356231, Corning, Corning, NY, USA) were used in this study. HuL001 (equal to HuL227) is HuniLife’s proprietary ENO1 mAb as described previously [26]. Human IgG1 antibodies (#HG1K, Sino Biological, Beijing, China) were used as an isotype control for HuL001. The concentrations of gemcitabine and HuL001 were selected based on preliminary optimization experiments together with supporting literature [26,27], whereas the concentrations of TXA and 2-DG were selected according to previously published studies [26,28,29,30].

### 2.6 Animal Studies

All animal studies were conducted in accordance with protocols approved by the Institutional Animal Care and Use Committee (IACUC) of TFBS Bioscience (IACUC protocol nos. TFBS2023-003, TFBS2024-002, and TFBS2025-002). A total of 104 female BALB/cAnN.Cg-Foxn1nu/CrlNarl (nude) mice, aged 6 to 8 weeks, and weighing 18–20 g, were obtained from the National Center for Biomodels, National Applied Research Laboratories, Taiwan, and used in this study. For all *in vivo* experiments, the experimental unit was a single mouse. Group sizes were determined based on prior experience with the PDAC xenograft models and the anticipated treatment effect size, and no formal power calculation was performed. No formal inclusion or exclusion criteria were established a priori for the animal studies, unless otherwise stated. Animals that died before the study endpoint were excluded only from endpoint-based analyses, and these exclusions are indicated in the relevant figure legends. All measurements were performed by investigators blinded to group allocation. 

The PANC-1/M and MIA PaCa-2 subcutaneous and orthotopic pancreatic models, together with the PDAC-TME mouse models, were used to approximate the tumor microenvironment and growth kinetics of PDAC. For the subcutaneous xenograft models using PANC-1/M and MIA PaCa-2 cells, cells were washed with PBS and resuspended in serum-free DMEM (#11995065, Gibco, Thermo Fisher Scientific) mixed 1:1 with Matrigel (#356231, Corning). A total of 3 × 10^6^ cells in 100 μL were implanted subcutaneously into the right flank of the mice. In the PANC-1/M dose-response subcutaneous model, mice were randomized into five groups (n = 5–6 per group), and treatments were administered intraperitoneally twice per week for 28 days, including vehicle, gemcitabine (20 mg/kg), and HuL001 at 5, 10, and 20 mg/kg. In the combination-therapy subcutaneous models, mice received PBS, HuL001, gemcitabine, or gemcitabine + HuL001 by intraperitoneal injection every 3 days, with n = 5 per group for the PANC-1/M model and n = 4 per group for the MIA PaCa-2 model. In these experiments, gemcitabine was administered at 20 or 50 mg/kg in the PANC-1/M model and at 20 mg/kg in the MIA PaCa-2 model, whereas HuL001 was administered at 20 mg/kg. For the PANC-1/M orthotopic model, 3 × 10^6^ cells mixed 1:1 with Matrigel (#356231, Corning) in a final volume of 40 μL were injected into the pancreatic tail of each nude mouse. Mice were assigned to PBS, HuL001, gemcitabine, or gemcitabine + HuL001 groups (n = 5 per group), and treatment began 7 days after inoculation. During the treatment period, mice were intraperitoneally injected every 3 days. In this model, HuL001 was administered at 30 mg/kg, and gemcitabine was administered at 10 mg/kg. For the PDAC-TME mouse models, PDAC-educated THP-1 cells were generated through direct co-culture of THP-1 cells with PANC-1/M cells at a 1:10 ratio, with or without HuL001 (100 μg/mL) treatment for 3 days before harvest. This tumor-cell-dominant condition was used to promote efficient monocyte education before subsequent *in vivo* co-implantation. A total of 3 × 10^6^ PANC-1/M cells were subcutaneously injected, with or without 3 × 10^5^ PDAC-educated THP-1 cells, into nude mice. Three groups were included in this model: PANC-1/M cells alone, PANC-1/M plus PDAC-educated THP-1 cells, and PANC-1/M plus HuL001-pretreated PDAC-educated THP-1 cells (n = 6 per group). The primary outcome measures were tumor volume and percentage tumor growth inhibition (%TGI). At study termination, animals were euthanized by CO_2_ inhalation in accordance with institutional guidelines.

### 2.7 Measurement of Viability of PDACs after Co-Culturing with Monocytes

A co-culture system of PDAC cells and monocytes was established using Transwell inserts (0.4 μm; #PTHT24H48, Merck Millipore, Burlington, MA, USA) placed in 24-well culture plates, as previously described in the study by Meng et al. [31] with minor modifications. Compared with the referenced protocol, our assay used a 0.4-μm Transwell system to assess paracrine interactions rather than the 8.0-μm migration format, and PANC-1/M cells and monocytes were co-cultured at a 1:1 ratio in complete DMEM (#11995065, Gibco, Thermo Fisher Scientific) instead of serum-free medium. This Transwell-based co-culture format was used to assess paracrine interactions between PDAC cells and monocytes while minimizing direct cell-cell contact. In contrast, the direct co-culture condition used to generate PDAC-educated THP-1 cells for the *in vivo* experiments was designed to facilitate tumor-cell-driven monocyte education and was therefore not intended for direct quantitative comparison with the Transwell assays. PANC-1/M cells (1 × 10^4^ in bottom well) were co-cultured with U937 or THP-1 cells (1 × 10^4^ in insert) in complete DMEM (#11995065, Gibco, Thermo Fisher Scientific), with 900 μL medium added to the lower chamber and 100 μL to the upper insert, and incubated at 37°C in 5% CO_2_, and simultaneously treated with 0, 1, 10, or 100 μg/mL of HuL001, or 100 μg/mL of control hIgG1 (#HG1K, Sino Biological), for up to 72 h. Subsequently, PANC-1/M cells were collected and assessed for viability using the Cell Counting Kit-8 (CCK-8; #CK04-20, Dojindo Laboratories, Kumamoto, Japan). For the CCK-8 assay, the inserts were first removed, and 100 μL CCK-8 reagent was added to each well, followed by incubation for 2 h at 37°C. The OD value at 450 nm was measured using a Thermo Scientific™ Multiskan Spectrum microplate reader (Thermo Fisher Scientific), operated with SkanIt™ Software (Thermo Fisher Scientific) for Microplate Readers, under light-protected conditions, which correlated proportionally with the number of viable cells, according to the manufacturer’s instructions. 

### 2.8 Reverse Transcription-Quantitative PCR (RT-qPCR)

PANC-1/M (2 × 10^5^) and MIA PaCa-2 (2 × 10^5^) cells were cultured in complete DMEM at 37°C in 5% CO_2_ and treated with 50 ng/mL TGFβ1 in the absence or presence of HuL001 at 1, 10, or 100 μg/mL, or 10 μg/mL control hIgG1 for 48 h. Subsequently, cells were collected, by aspirating the culture medium, washing once with PBS, and lysing the cells directly in the culture wells for RNA extraction. A co-culture system of PDAC cells with monocytes was established using transwell inserts (0.4 μm; #PTHT24H48; Merck Millipore), which were placed in 24-well culture plates. PANC-1/M (2 × 10^5^) cells were seeded into the inserts, while U937 or THP-1 cells (2 × 10^5^) were placed in the lower compartment of the culture system for 3 days. Subsequently, the U937 or THP-1 cells were collected by gentle pipetting, washed once with PBS, and processed immediately for RNA extraction. Total RNA from treated cells was extracted using an rSYNC RNA Isolation Kit (#RS300, Geneaid Biotech Ltd., New Taipei City, Taiwan) according to the manufacturer’s instructions, including the recommended on-column DNase removal step when applicable. RNA concentration and purity were determined spectrophotometrically before reverse transcription. Reverse transcription (RT) was performed using the iScript™ cDNA Synthesis Kit (#1708891, Bio-Rad Laboratories, Hercules, CA, USA) with equal amounts of RNA input for each sample, according to the manufacturer’s instructions. The primer sequences used for cDNA amplification were listed in Table 1. Quantitative PCR (qPCR) was performed on a Bio-Rad CFX384 using the PowerUp™ SYBR™ Green Master Mix (Applied Biosystems, Thermo Fisher Scientific). Each reaction was carried out in a total volume of 10 μL, containing 1.5 μL diluted cDNA (10 ng/μL; 15 ng input per reaction), 5 μL 2× master mix, 0.5 μL each of forward and reverse primers (10 μM; final concentration, 500 nM each), and 2.5 μL ddH_2_O. The following thermal cycling conditions were used: 50°C for 2 min; 95°C for 2 min; then 40 cycles of 95°C for 15 s and 60°C for 1 min. Fold changes in target gene expression were calculated using the relative quantification method. ΔCq values were obtained as follows: ΔCq = Cq of ACTB − Cq of the target gene. ΔΔCq values were then calculated as: ΔΔCq = ΔCq of the treated group − ΔCq of the control group. Fold change was calculated as 2^−^^ΔΔCq^, with control groups normalized to 1-fold.

**Table 1:** Primer sequences used for Quantitative real-time PCR.

Gene	Forward (5′ → 3′)	Reverse (5′ → 3′)
*HK2*	GAGTTTGACCTGGATGTGGTTGC	CCTCCATGTAGCAGGCATTGCT
*IL10*	TCTCCGAGATGCCTTCAGCAGA	TCAGACAAGGCTTGGCAACCCA
*TGFB1*	TACCTGAACCCGTGTTGCTCTC	GTTGCTGAGGTATCGCCAGGAA
*VEGFA*	TTGCCTTGCTGCTCTACCTCCA	GATGGCAGTAGCTGCGCTGATA
*ENO1*	AGTCAACCAGATTGGCTCCGTG	CACAACCAGGTCAGCGATGAAG
*ACTB*	CACCATTGGCAATGAGCGGTTC	AGGTCTTTGCGGATGTCCACGT

### 2.9 Flow Cytometry Detection of Cell Surface ENO1

PANC-1, PANC-1/M, and MIA PaCa-2 cells were incubated with Fc receptor block (#130-059-901, Miltenyi Biotech, Bergisch Gladbach, Germany) for 10 min at 4°C, then washed once in cold Stain Buffer (#554656, BD Biosciences, Franklin Lakes, NJ, USA) at 300× *g* for 5 min at RT. Cells were resuspended at 1 × 10^6^ cells/tube in 100 μL of cold Stain Buffer and stained with APC-conjugated anti-ENO1 antibody (#ab305881, Abcam, dilution 1:200) or isotype-matched rabbit IgG1 control (#ab232814, Abcam, dilution 1:200) diluted in the same buffer for 60 min in the dark on ice, then washed twice with cold Stain Buffer at 300× *g* for 5 min at RT. After staining, samples were resuspended in cold Stain Buffer and analyzed using a CytoFlex V0-B4-R2 flow cytometer (Beckman Coulter, Brea, CA, USA). Data were acquired with CytExpert 2.4 software (Beckman Coulter) and analyzed using Kaluza Analysis 2.1 software (Beckman Coulter). For each sample, 1 × 10^4^ cells were acquired. The percentage of ENO1-positive surface cells was determined by subtracting the percentage of cells stained with the isotype control from that of the ENO1-stained samples.

### 2.10 Measurement of Secreted ENO1, Lactate, and Cytokines

The levels of secreted ENO1, lactate, and cytokines in the cell culture supernatant or tumor tissue lysates were measured using commercially available ELISA or colorimetric assay kits, following the manufacturers’ protocols, and absorbance was read using a Thermo Scientific™ Multiskan Spectrum microplate reader operated with SkanIt™ Software for Microplate Readers (Thermo Fisher Scientific). Specifically, ENO1 was quantified using the Human ENO1 ELISA Kit (Alpha-Enolase; #ab181417, Abcam), lactate using a colorimetric assay kit (#MET-5012, Cell Biolabs, San Diego, CA, USA), and TGFβ1 using a human ELISA kit (#DY240-05, R&D Systems, Minneapolis, MN, USA).

### 2.11 Measurement of Plasminogen Receptor Activity of ENO1

PANC-1/M cells, stimulated with or without 50 ng/mL human TGFβ1 (#100-21, PeproTech), were incubated at 37°C for 24 h, then harvested, washed twice with PBS at 300× *g* for 2 min at RT, and resuspended in PBS at a concentration of 1 × 10^6^ cells/mL. The cells were preincubated at 37°C for 3 h with or without HuL001 (1, 10, 100 μg/mL) and hIgG1 (100 μg/mL). Following this, they were treated with 40 μM human Glu-plasminogen (#528180, Sigma-Aldrich, St. Louis, MO, USA) for 1 h. After incubation, the cells were washed three times with PBS and resuspended in 100 μL PBS containing 15 nM tissue plasminogen activator (tPA; #10157-HNCH2, Sino Biological) and 1 mM plasmin chromogenic substrate, Chromogenix S-2251 (#S820332, DiaPharma, West Chester, OH, USA), and incubated at 37°C for 3 h. Plasminogen receptor activity (plasmin activation) was assessed by measuring absorbance at 405 nm using a Thermo Scientific™ Multiskan Spectrum microplate reader (Thermo Fisher Scientific), operated with SkanIt™ Software (Thermo Fisher Scientific) for Microplate Readers.

### 2.12 Statistical Analysis

Results are presented as the mean ± standard deviation (SD) from at two independent experiments or as the mean ± standard error of the mean (SEM) of biological replicates, as specified in each figure legend. Statistical analyses were performed using GraphPad Prism 8 (GraphPad Software, San Diego, CA, USA). The Student’s *t*-test was used to compare differences between two groups. Data normality and variance homogeneity were routinely evaluated, and when these assumptions were not met, appropriate non-parametric tests were applied. Statistical significance was defined as *p* < 0.05 and is indicated as *p < 0.05; **p < 0.01, and ***p < 0.001. 

## 3 Results

### 3.1 Elevated Surface Expression of ENO1 Correlates with Advanced Disease Stage and Reduced Survival in PDAC Patients

**Figure 1:**
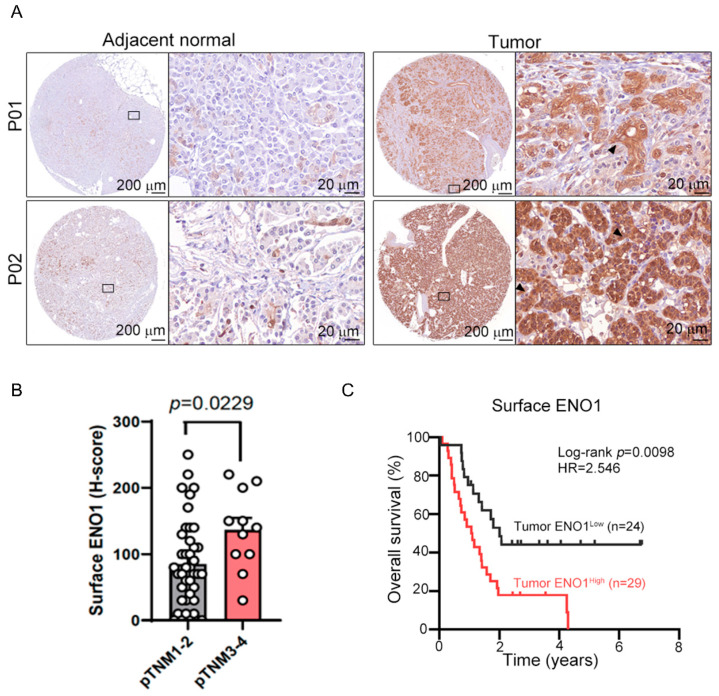
**Higher surface α-enolase (ENO1) expression correlated with worse overall survival in patients with pancreatic ductal adenocarcinoma (PDAC).** (**A**) Representative IHC images of surface ENO1 expression in adjacent normal tissue and tumor tissue from two PDAC patients (P01 and P02). The black boxed regions in the inset (low-magnification view) indicate the corresponding high-magnification field. The arrowheads indicate areas of positive ENO1 staining in the tumor tissue. The scale bar represents 20 μm. (**B**) Quantification of surface ENO1 expression using H-score in PDAC patient samples, stratified by pTNM stage (pTNM1-2 and pTNM3-4). (**C**) Kaplan-Meier survival curves for PDAC patients with high and low surface ENO1 expression. Patients were stratified into high and low ENO1 expression groups based on the median H-score. Overall survival (OS) was calculated from the date of diagnosis to the date of death or last follow-up. Statistical significance was determined using the log-rank test. The hazard ratio (HR) and 95% confidence interval (CI) are indicated.

To investigate the clinical significance of surface ENO1 expression in tumor tissues, we performed IHC analysis on paired tumors and adjacent normal tissues from patients diagnosed with PDAC (Fig. 1A). Strong membrane-associated ENO1 staining was observed in tumor samples, compared to adjacent normal tissues which exhibited minimal ENO1 staining. Representative images from two patients (P01 and P02) clearly demonstrate elevated ENO1 immunoreactivity in tumor cells, with prominent membranous staining (black arrowheads). Quantitative analysis of ENO1 surface expression using H-score revealed a statistically significant increase in tumors classified as stage pTNM3-4 compared to those in stage pTNM1-2 (Fig. 1B). The mean H-score was significantly higher in advanced-stage tumors, suggesting that ENO1 surface expression is associated with tumor progression. We next assessed the prognostic value of ENO1 surface expression by stratifying patients into high and low ENO1 groups based on IHC H-scores. Kaplan–Meier survival analysis demonstrated that patients with high surface ENO1 expression exhibited significantly shorter overall survival (OS) in contrast to those with low ENO1 levels (Fig. 1C). The hazard ratio (HR) for death in the high ENO1 group was 2.546 (95% CI: 1.339–4.848), indicating a more than two-fold increased risk of mortality associated with the elevated ENO1 expression. These results collectively suggest that surface ENO1 expression is upregulated in tumor tissues and correlates with both advanced pathological stage and reduced overall survival, underscoring its potential as a prognostic biomarker and therapeutic target in PDAC.

### 3.2 ENO1-Targeting Monoclonal Antibody (mAb) HuL001 Suppresses Tumor Growth in a Dose-Dependent Manner in a PANC-1/M Xenograft Model

**Figure 2:**
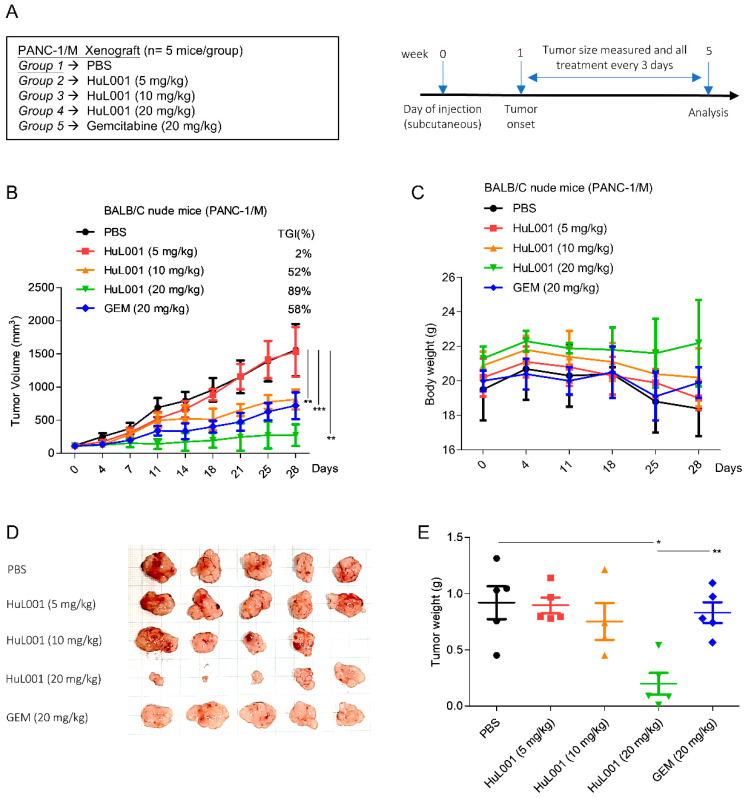
**HuL001 inhibited the tumor growth of PDAC in a dose-responsive manner.** (**A**) Experimental design: Nude mice were subcutaneously implanted with PANC-1/M cells on the flank, and treatment started when the tumors reached a mean volume of 107 mm^3^ (set as Day 0). Mice were randomized into five groups as indicated. Treatments of HuL001 or GEM were administered intraperitoneally (i.p.) twice per week for a total of 28 days. (**B**) Tumor volume was measured every 3–4 days, and the graph shows the average tumor volume for each group over time. (**C**) Body weight was measured weekly. At the end of the study, the tumors were harvested with the images taken (**D**), and the tumor weight (**E**) was measured. Positions with no tumor sample indicate mice that died prior to the study endpoint and were excluded from the endpoint analysis. Each data point represents the mean ± standard error of the mean (SEM) of biological replicates (n = 5–6 per group). Symbols: *p < 0.05, **p < 0.01, ***p < 0.001.

Given the association between elevated surface ENO1 expression and advanced disease stage in PDAC (Fig. 1), we next evaluated the therapeutic potential of the ENO1-targeting mAb HuL001 *in vivo*. A subcutaneous xenograft model was established using PANC-1/M cells in immunodeficient nude mice, and treatment commenced when tumors reached a mean volume of approximately 100 mm^3^. Mice were randomized into five groups receiving vehicle (PBS), gemcitabine (standard of care), or escalating doses of HuL001 administered intraperitoneally twice weekly for 28 days (Fig. 2A). HuL001 significantly inhibited tumor growth in a dose-dependent manner, with the highest dose group showing marked suppression of tumor progression relative to both the vehicle and gemcitabine-treated groups (Fig. 2B). Notably, tumor volume in animals was reduced by treatment with HuL001 in a dose-responsive manner. The high-dose HuL001 has a superior anti-tumor efficacy (effect size = 1285 mm^3^, 95% CI = 843–1725, TGI = 89%, *p* = 0.0001) compared to that of gemcitabine at 20 mg/kg (effect size = 838 mm^3^, 95% CI = 381–1296, TGI = 58%, *p* = 0.0029). Treatment with HuL001 was well tolerated across all dose levels, with no significant changes in body weight, indicating an acceptable safety profile (Fig. 2C). At study termination, tumors were harvested and visibly smaller in the HuL001-treated animals compared to controls and gemcitabine (Fig. 2D), and tumor weights were significantly reduced in the HuL001-treated animals at 20 mg/kg (Fig. 2E). These findings demonstrate the *in vivo* efficacy of HuL001 and further support its potential as a therapeutic alternative or adjunct to current PDAC treatments.

### 3.3 HuL001 Attenuates PDAC Cell Proliferation and M2 Macrophage-Associated Cytokine Gene Expression in PDAC-Monocyte Co-Cultures

**Figure 3:**
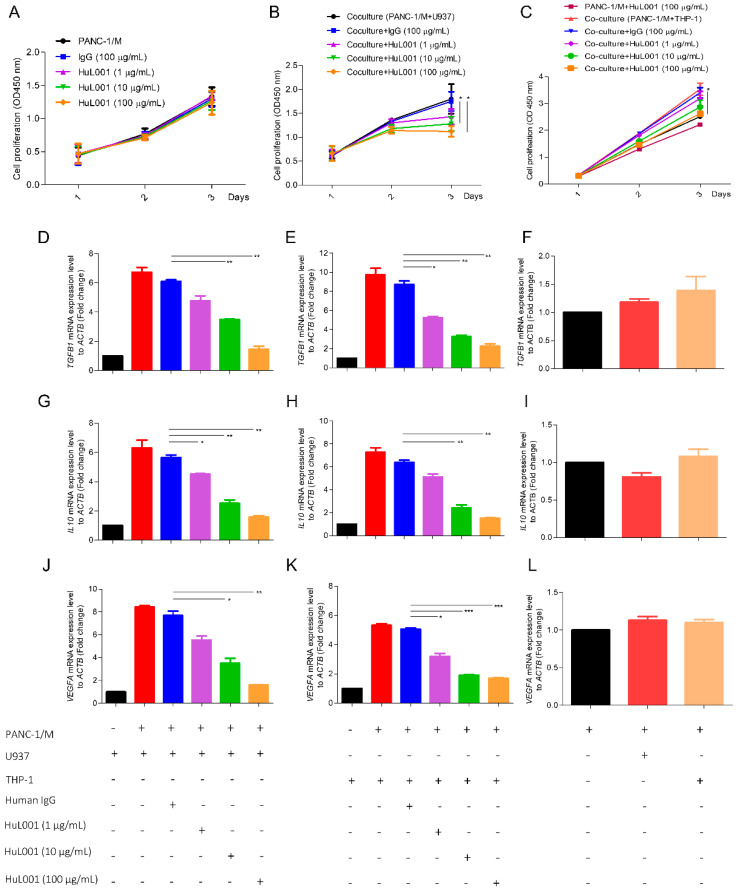
**HuL001 diminished monocyte-supported PDAC cell growth and reduced M2-associated cytokine mRNA expression in the PDAC-monocyte co-cultures.** PANC-1/M cells were cultured either alone (**A**) or co-cultured with U937 (**B**,**D**,**F**,**G**,**I**,**J**,**L**) or THP-1 (**C**,**E**,**F**,**H**,**I**,**K**,**L**) cells at a 1:1 ratio using a Transwell system with 0.4 μm pore-size inserts. For co-culture conditions, U937 or THP-1 cells were seeded in the transwell, while PANC-1/M cells were cultured in the bottom well. The cells were treated with HuL001 at 1, 10, or 100 μg/mL or human IgG (100 μg/mL). The proliferation of PANC-1/M cells alone (**A**) or co-cultured with U937 (**B**) or THP-1 (**C**) were assessed at days 1, 2, and 3 using the Cell Counting Kit-8 assay. The *TGFB1* mRNA levels in U937 (**D**), THP-1 (**E**), and PANC-1/M (**F**) cells, the *IL10* mRNA levels in U937 (**G**), THP-1 (**H**), and PANC-1/M (**I**) cells, and the *VEGFA* mRNA levels in U937 (**J**), THP-1 (**K**), and PANC-1/M (**L**) cells were quantified by RT-qPCR after 72 h of treatment. Relative expression was normalized to *ACTB* and presented as fold change compared to PANC-1/M cells alone. All data are represented as the mean ± SD of two independent experiments. Symbols: *p < 0.05, **p < 0.01, ***p < 0.001.

We first examined if HuL001 had a direct effect on cell growth of PANC-1/M cells, while no impact was observed in PANC-1/M cells cultured alone, comparable to the control of human IgG (Fig. 3A). To investigate the possible involvement of stromal components, we established a Transwell-based PDAC cells (PANC-1/M) and monocyte (U937 or THP-1) co-culture system to mimic the paracrine interactions in the tumor stroma. Given the role of TAMs in promoting PDAC progression via immune suppression and extracellular matrix remodeling [32], we examined whether HuL001 could modulate this pro-tumorigenic axis in co-cultures. While HuL001 had minimal impact on the proliferation of PANC-1/M cells cultured alone (Fig. 3A), treatment at escalating concentrations (1, 10, or 100 μg/mL) significantly and dose-dependently reduced PANC-1/M cell proliferation over a 3-day period when co-cultured with U937 monocytes (Fig. 3B), or with THP-1 monocytes (Fig. 3C). This differential response highlights the importance of monocyte-derived pro-tumorigenic signals in driving PDAC cell growth and suggests that HuL001 effectively targets tumor-host interactions rather than direct cytotoxicity. We next analyzed the expression of *TGFB1*, *IL10*, and *VEGFA*, key cytokines associated with M2-like macrophage polarization and known contributors to immune evasion and tumor progression in PDAC [32]. For *TGFB1*, co-culture with U937 or THP-1 monocytes induced marked upregulation in monocytes, which was significantly suppressed by HuL001 in a dose-dependent manner (Fig. 3D,E), whereas only slight changes were observed in PANC-1/M cells under the same co-culture conditions (Fig. 3F). A similar pattern was observed for *IL10*, with robust induction in U937 and THP-1 monocytes and suppression by HuL001 (Fig. 3G,H), but only slight increases in PANC-1/M cells (Fig. 3I). Likewise, *VEGFA* expression was strongly induced in U937 and THP-1 monocytes and attenuated by HuL001 (Fig. 3J,K), whereas only modest changes were detected in PANC-1/M cells (Fig. 3L), suggesting that monocytes were the major contributors to the immunosuppressive cytokine response in this co-culture system. These results suggest that HuL001 not only counteracts monocyte-driven tumor cell proliferation but also interferes with the establishment of an immunosuppressive cytokine milieu characteristic of the PDAC microenvironment.

### 3.4 HuL001 Suppresses Plasmin-Mediated Activation of TGFβ in PDAC-Monocyte Co-Cultures

**Figure 4:**
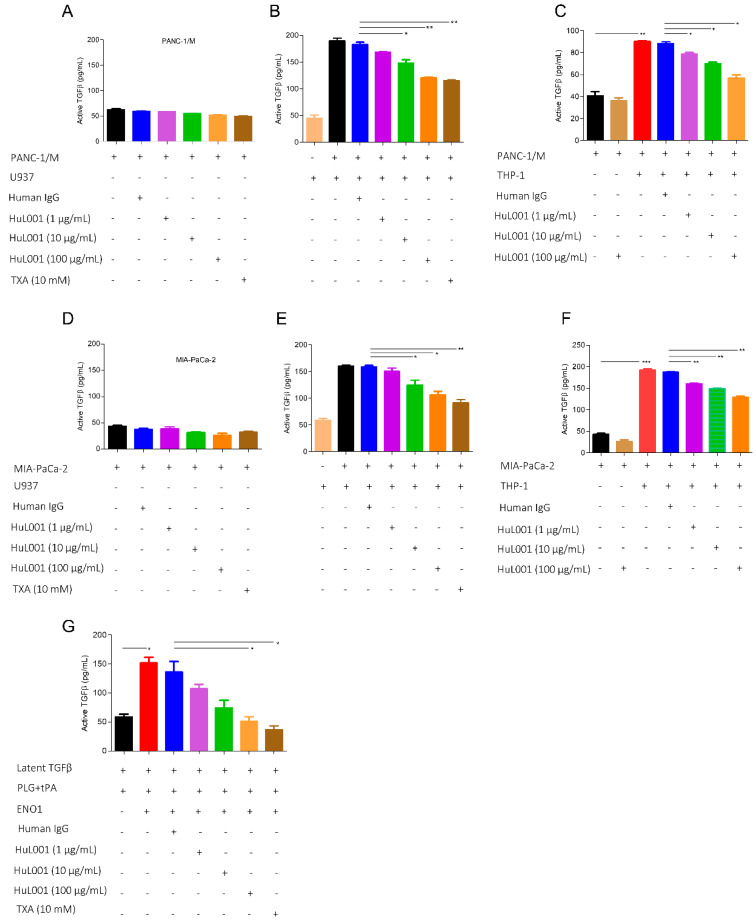
**HuL001 attenuated ENO1/plasmin-dependent activation of transforming growth factor β (TGFβ) in PDAC-monocyte co-cultures.** PANC-1/M cells were cultured separately (**A**) or co-cultured at a 1:1 ratio with U937 cells (**B**) or THP-1 cells (**C**) in a Transwell system with 0.4-μm pore-size inserts. MIA PaCa-2 cells were cultured separately (**D**) or co-cultured at a 1:1 ratio with U937 cells (**E**) or THP-1 cells (**F**) under the same conditions. For co-culture conditions, U937 (**B**,**E**) or THP-1 cells (**C**,**F**) were seeded in the transwell, while PANC-1/M or MIA PaCa-2 cells were cultured in the bottom well. The cells were treated with HuL001 at 1, 10, or 100 μg/mL, control IgG (100 μg/mL), or tranexamic acid (TXA) (10 mM) for 72 h. The culture supernatants were collected for active TGFβ quantification (**A**–**F**). (**G**) Plasminogen (40 μM), tPA (15 nM), and latent TGFβ (20 ng/mL) were incubated at 37°C for 3 h with or without ENO1 (4 μg/mL), control IgG (100 μg/mL), HuL001 (1, 10, or 100 μg/mL), or TXA (10 mM), followed by the quantification of active TGFβ. Results are represented as the mean ± SD of two independent experiments. Symbols: *p < 0.05, **p < 0.01, ***p < 0.001.

Among the M2 macrophage-associated cytokines suppressed by HuL001 (Fig. 3), TGFβ is particularly critical in PDAC due to its established roles in immune suppression and stromal remodeling [33]. To investigate whether HuL001 modulates TGFβ activation in the PDAC TME, we first assessed the effects of HuL001 on the generation of active TGFβ in PDAC and monocyte cultures. In non-co-cultured PANC-1/M cells, HuL001 led to a modest but measurable reduction in active TGFβ levels compared to IgG controls (Fig. 4A). Tranexamic acid (TXA) is a plasmin inhibitor. It acts as a synthetic lysine analogue that blocks plasminogen’s ability to bind to its receptor, ENO1. TXA at 10 mM also inhibited TGFβ activation, suggesting such inhibition might be related to plasmin protease activity. Furthermore, in a Transwell-based co-culture system, where PANC-1/M cells and U937 (or THP-1) monocytes were physically separated to allow paracrine communication, HuL001 markedly suppressed the accumulation of active TGFβ in the conditioned media in a concentration-dependent manner (Fig. 4B,C). The magnitude of inhibition by HuL001 was comparable to that achieved with plasmin inhibitor TXA, implicating that ENO1 was in the upstream regulation of plasmin-mediated TGFβ activation via blocking the plasminogen interaction to its receptor ENO1 and thus the generation of plasmin (Fig. 4A,B). As shown by the first bar in Fig. 4B, active TGFβ levels were also measured in U937 cells cultured alone, which served as an additional single-cell control. PANC-1/M was used as the primary mechanistic and co-culture model, whereas MIA PaCa-2 was included for complementary validation as a second PDAC background. Similar inhibitory effects of HuL001 on active TGFβ accumulation were also observed in MIA PaCa-2 cells under both non-co-cultured conditions and Transwell-based co-culture conditions with U937 or THP-1 monocytes (Fig. 4D–F), thereby providing complementary validation of the findings obtained in PANC-1/M cells. To confirm the involvement of the ENO1-plasmin-TGFβ axis, we measured active TGFβ by using a cell-free TGFβ activation assay containing latent TGFβ, plasminogen, and tissue plasminogen activator (tPA). The presence of recombinant ENO1 as a plasminogen receptor markedly enhanced the conversion of latent to active TGFβ, which was effectively blocked by HuL001 in a dose-dependent manner, comparable to the level of active TGFβ upon TXA treatment (Fig. 4G). These findings support a model in which tumor-rich ENO1 promotes plasmin generation and subsequent conversion of latent TGFβ to its active form in the TME. 

### 3.5 HuL001 Attenuates the Pro-Tumorigenic Activity of PDAC-Educated Monocytes In Vivo

**Figure 5:**
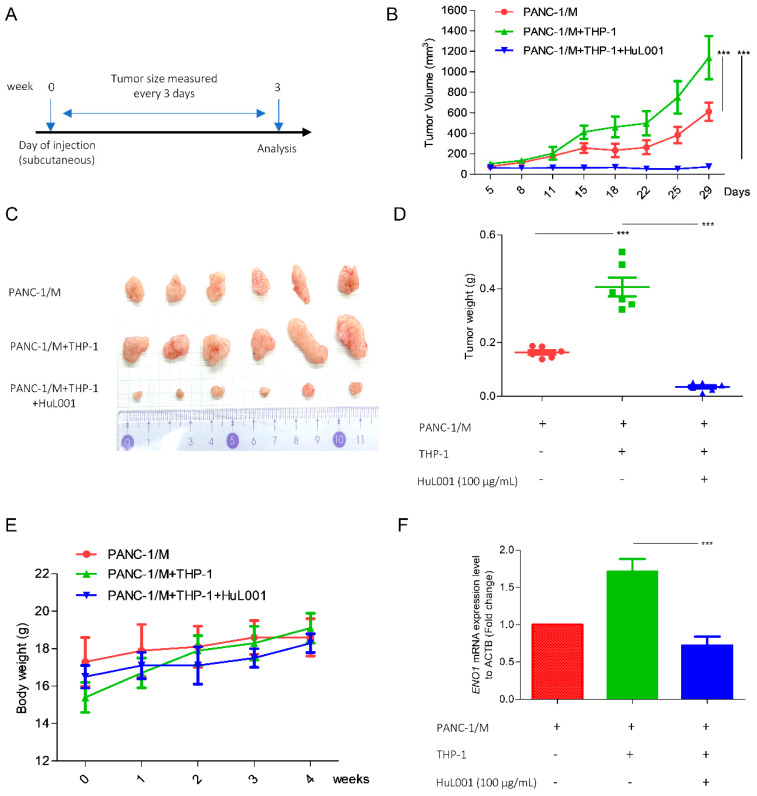
**HuL001 pretreatment attenuated the tumor-promoting activity of PDAC-educated monocytes *in vivo*.** Prior to injection, PANC-1/M cells were cultured alone, or directly co-cultured with THP-1 monocytes at a 10:1 ratio with or without HuL001 (100 μg/mL) for 72 h. After co-culture, adherent PANC-1/M cells and suspension PDAC-educated THP-1 cells were harvested separately. Three groups of cells were prepared for subcutaneous injection into nude mice: PANC-1/M cells alone, PANC-1/M mixed with PDAC-educated THP-1 cells (10:1), and HuL001-pretreated mixture of PANC-1/M and PDAC-educated THP-1 cells (10:1). (**A**) Schematic overview of the experimental design. (**B**) Tumor volume was measured every 3 days and plotted over time. (**C**) Representative tumor images at the study endpoint. (**D**) Tumor weights at the endpoint. (**E**) Body weight was monitored weekly. (**F**) *ENO1* mRNA levels in six representative tumors per group were quantified by RT-qPCR. Expression was normalized to *ACTB* and presented as fold change relative to the PANC-1/M alone group. Each data point is presented as the mean ± SEM of biological replicates (n = 6 per group). Symbols: ***p < 0.001.

Building upon our findings *in vitro* that HuL001 inhibits monocyte-induced PDAC cell proliferation (Fig. 3B,C), we next investigated the effect of HuL001 on the tumor-promoting activity of PDAC-educated monocytes *in vivo* (Fig. 5A). Prior to cell implantation in animals, cell expansions were derived from PANC-1/M cells or mixed cultures of PANC-1/M and THP-1 cells (in the presence or absence of HuL001). As expected, co-injection of PDAC-educated THP-1 cells significantly enhanced tumor growth compared to PANC-1/M cells alone in animals (Fig. 5B), confirming the acquisition of a tumor-promoting phenotype by monocytes. Representative tumor images at endpoint supported these findings as well (Fig. 5C). Importantly, HuL001 pretreatment *in vitro* only during co-culture markedly suppressed this tumor-promoting effect, leading to significantly reduced tumor volumes throughout the study period and lower final tumor weights (Fig. 5D). No significant differences in body weight were observed between groups (Fig. 5E), suggesting that HuL001 treatment did not induce systemic toxicity. In addition, co-injection with PDAC-educated THP-1 cells significantly increased *ENO1* expression in tumor tissues compared with tumors derived from PANC-1/M cells alone (Fig. 5F), whereas HuL001 pretreatment during the *in vitro* co-culture phase significantly attenuated this increase. These findings further support the involvement of ENO1 in monocyte-mediated tumor-promoting activity *in vivo* and indicate that HuL001 impairs the tumor-promoting capability of PDAC-educated monocytes.

### 3.6 HuL001 Blocks Plasmin Activity and Cell Proliferation Driven by the ENO1-TGFβ Axis in PDAC

**Figure 6:**
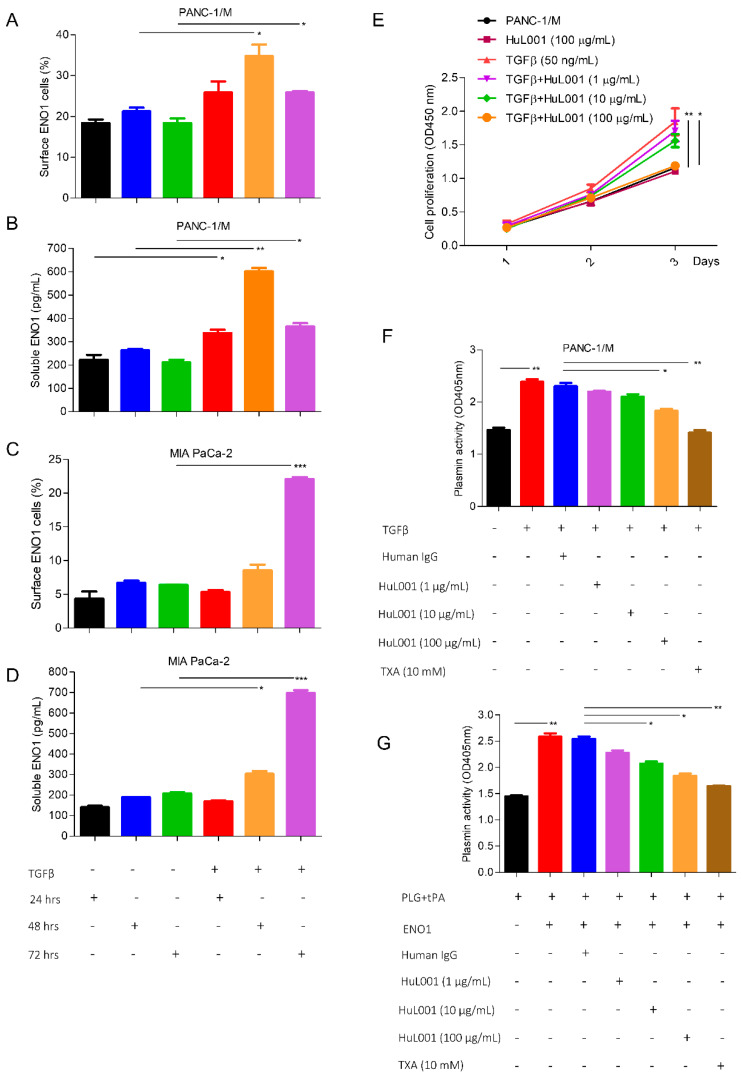
HuL001 inhibited plasmin activity induced by ENO1 or TGFβ and suppressed TGFβ-induced cell proliferation in PDAC cells. PANC-1/M (**A**,**B**) and MIA PaCa-2 (**C**,**D**) were treated with TGFβ (50 ng/mL) for 24, 48, and 72 h. These cells were then harvested for surface ENO1 detection by using flow cytometry (**A**,**C**), and cell-free supernatants were collected for soluble ENO1 quantification (**B**,**D**). (**E**) PANC-1/M cells were treated with HuL001 alone (100 μg/mL), TGFβ alone (50 ng/mL), and TGFβ combined with different concentrations of HuL001 (1, 10, and 100 μg/mL), and cell proliferation was assessed on day 1, 2, or 3 using the Cell Counting Kit-8 assay. (**F**) PANC-1/M cells were treated with TGFβ (50 ng/mL) alone or in combination with HuL001 (1, 10, and 100 μg/mL), IgG1 (100 μg/mL), and TXA (10 mM) for 24 h, followed by measurement of plasmin activity. (**G**) Plasminogen (40 μM) and tPA (15 nM) were added with or without ENO1 (4 μg/mL), IgG1 (100 μg/mL), HuL001 (1, 10, or 100 μg/mL), and TXA (10 mM) at 37°C for 3 h, followed by measurement of plasmin activity. All data are represented as the mean ± SD from two independent experiments. Symbols: *p < 0.05, **p < 0.01, ***p < 0.001.

Given the role of ENO1 in facilitating plasmin-mediated TGFβ activation (Fig. 4) and the tumor-promoting crosstalk observed in the PDAC-monocyte axis (Fig. 5), we next investigated whether TGFβ stimulation enhanced ENO1 expression and its ensuing plasmin activity in PDAC cells. Upon TGFβ treatment, both surface-associated and soluble ENO1 levels were markedly upregulated in PANC-1/M cells 48 h post-stimulation (Fig. 6A,B) and in MIA PaCa-2 cells 72 h post-stimulation (Fig. 6C,D). Functionally, TGFβ stimulation enhanced proliferation of PANC-1/M cells over 72 h, which was attenuated by co-treatment with HuL001 in a dose-dependent manner (Fig. 6E). Consistent with this, TGFβ treatment increased plasmin activity in PANC-1/M cells, which was significantly suppressed by HuL001 or by TXA as well, but not by control IgG (Fig. 6F). These results also suggest that TGFβ could be one of the critical components generated from the co-culture of PDAC and monocytes to induce PDAC cell proliferation observed in Fig. 3B,C. Furthermore, in a cell-free reconstitution assay, recombinant ENO1 markedly enhanced plasmin generation in the presence of plasminogen and tPA, whereas HuL001 dose-dependently reduced this effect (Fig. 6G). These findings confirm a feed-forward loop in which TGFβ upregulates ENO1 expression, which in turn enhances plasmin generation and promotes further TGFβ activation. HuL001 disrupts this loop at multiple levels, supporting its role as a mechanistic inhibitor of the ENO1-plasmin-TGFβ axis in PDAC.

### 3.7 HuL001 Suppresses TGFβ-Induced Glycolytic Activity in PDAC Cells

**Figure 7:**
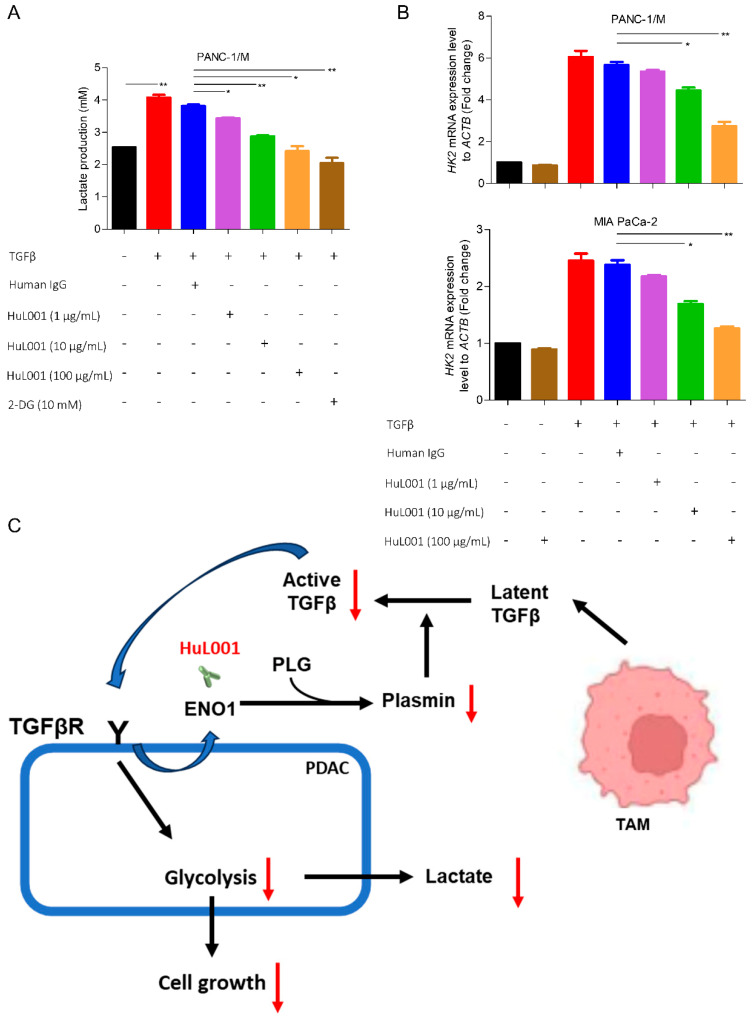
HuL001 reduced TGFβ-induced glycolytic activity (HK2 gene expression and lactate production) in PDAC cells. PANC-1/M (**A**,**B (upper panel)**) and MIA PaCa-2 (**B (lower panel)**) cells were treated with TGFβ (50 ng/mL) with or without HuL001 (1, 10, or 100 μg/mL), control IgG (100 μg/mL), 2-DG (10 mM) for 48 h. (**A**) The culture supernatants were collected to measure lactate concentrations. (**B**) The *HK2* mRNA levels were quantified by RT-qPCR. Relative expression was normalized to β-actin and presented as fold change compared to untreated controls. Results are represented as the mean ± SD of two independent experiments. Symbols: *p < 0.05, **p < 0.01. (**C**) Conceptual diagram illustrates the role of ENO1 in the PDAC microenvironment. ENO1 enhances plasmin generation, activating latent TGFβ produced from TAMs, which signals through TGFβR, thereby enhancing lactate production (i.e., glycolysis), promoting cell growth, and facilitating the export of ENO1 to the cell surface and the extracellular space. This forms a feed-forward loop that sustains ENO1 expression and TGFβ signaling. Red arrows indicate HuL001-mediated downregulation.

Given that TGFβ signaling can promote glycolysis [34], we next investigated whether ENO1 contributes to TGFβ-driven metabolic reprogramming in PDAC cells. The TGFβ-induced glycolytic activity, indicated by lactate production (Fig. 7A) and glycolytic enzyme HK2 gene expression (Fig. 7B), was attenuated by HuL001 and 2-DG in PDAC cell lines, but not by control IgG. A conceptual diagram (Fig. 7C) illustrates a feed-forward loop of ENO1-TGFβ-ENO1 whereas ENO1 enhances plasmin-dependent TGFβ activation, which in turn promotes tumor growth through glycolysis and sustains ENO1 expression via surface translocation. HuL001 disrupts this loop at multiple points, reducing both metabolic programming and proliferative signaling. 

### 3.8 HuL001 Enhances the Therapeutic Efficacy of Gemcitabine in PDAC Xenograft Models by Reducing Tumor Growth, Lactate Production, and TGFβ Activation

**Figure 8:**
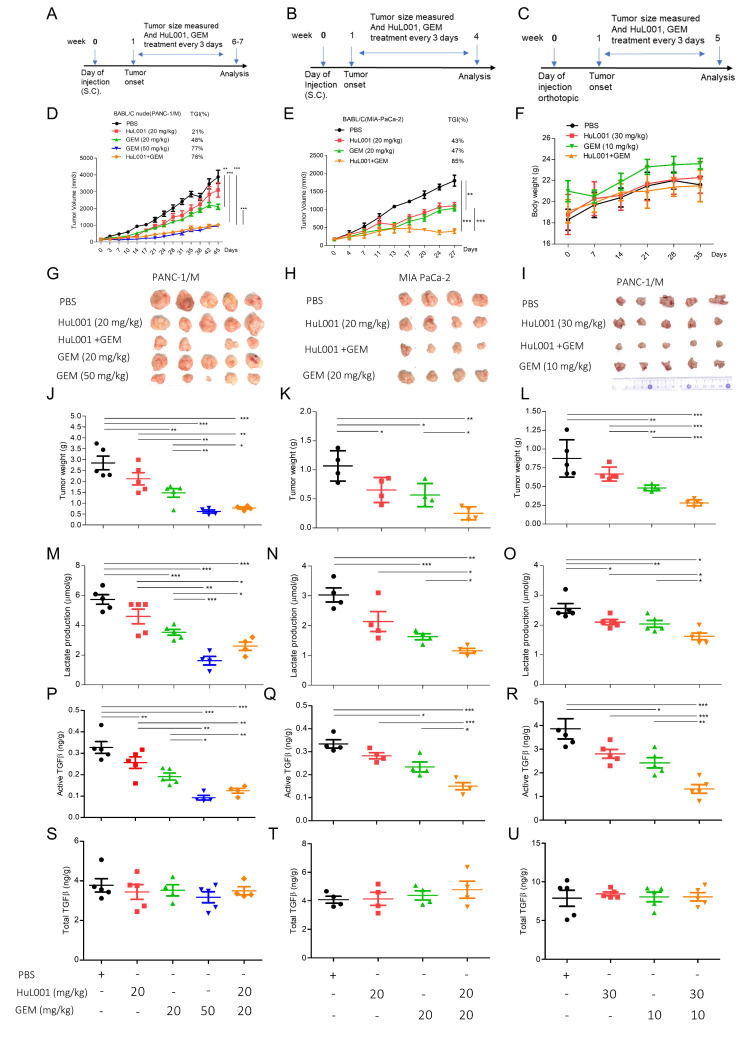
HuL001 monotherapy or combination therapy with GEM inhibited tumor growth, lactate production, and TGFβ activation in PDAC xenograft models. (**A**–**C**) A schematic timeline was shown for the experiment design. Nude mice were subcutaneously implanted with (**A**) PANC-1/M cells (n = 5 per group) or (**B**) MIA PaCa-2 cells (n = 4 per group) in the flank region. Treatment was initiated when the tumors reached an average volume of 156 mm^3^ for the PANC-1/M model and 172 mm^3^ for the MIA PaCa-2 model, which was designated as Day 0. (**C**) For the orthotopic model, nude mice (n = 5 per group) were injected with PANC-1/M cells into the pancreatic tail, and treatment began 7 days after inoculation. During the treatment period, mice were intraperitoneally injected with PBS (vehicle control), HuL001, gemcitabine (GEM), or GEM + HuL001 at the indicated concentrations every 3 days. Tumor volume was measured over time in the subcutaneous PANC-1/M model (**D**) and the subcutaneous MIA PaCa-2 model (**E**). Body weight was monitored in the orthotopic PANC-1/M model (**F**). The %TGI at the study endpoint was calculated for the indicated treatment groups. Representative tumor images from the subcutaneous PANC-1/M, subcutaneous MIA PaCa-2, and orthotopic PANC-1/M models are shown in (**G**–**I**), respectively. Tumor weights from the subcutaneous PANC-1/M, subcutaneous MIA PaCa-2, and orthotopic PANC-1/M models are shown in (**J**–**L**), respectively. Lactate production in tumor tissues from the subcutaneous PANC-1/M, subcutaneous MIA PaCa-2, and orthotopic PANC-1/M models is shown in (**M**–**O**), respectively. Active TGFβ1 levels in tumor tissues from the subcutaneous PANC-1/M, subcutaneous MIA PaCa-2, and orthotopic PANC-1/M models are shown in (**P**–**R**), respectively. Total TGFβ levels (inactive and active forms) in tumor tissues from the subcutaneous PANC-1/M, subcutaneous MIA PaCa-2, and orthotopic PANC-1/M models are shown in (**S**–**U**), respectively. Treatments were administered for 4–7 weeks, after which the mice were sacrificed, and tumor tissues and plasma samples were collected. Positions with no tumor sample indicate mice that died prior to the study endpoint and were excluded from the endpoint analysis. Each data point is presented as the mean ± SEM. * *p* < 0.05; ** *p* < 0.01; *** *p* < 0.001.

To evaluate the potential of HuL001 as a combination partner to standard chemotherapy, we assessed its therapeutic effect together with gemcitabine (GEM) in three PDAC xenograft models (Fig. 8A–C): subcutaneous PANC-1/M, subcutaneous MIA PaCa-2, and orthotopic PANC-1/M. Treatment began when tumors reached ~100 mm^3^ for the subcutaneous model or 7 days post-inoculation for the orthotopic model. In the subcutaneous PANC-1/M model, HuL001 combined with GEM at 20 mg/kg achieved a TGI of 78%, outperforming both GEM monotherapy at 20 mg/kg (effect size = 1777 mm^3^, 95% CI = 791–2764, TGI = 48%, *p* = 0.0032) and even at 50 mg/kg (effect size = 2925 mm^3^, 95% CI = 1845–4007, TGI = 77%, *p* = 0.0004) (Fig. 8D). In the MIA PaCa-2 model, the combination conferred superior antitumor effects (effect size = 1401 mm^3^, 95% CI = 997–1805, TGI = 85%, *p* = 0.0001) compared to GEM alone at 20 mg/kg (effect size = 765 mm^3^, 95% CI = 348–1181, TGI = 47%, *p* = 0.0041) (Fig. 8E). Body weight remained stable across treatment groups, suggesting good tolerability in the orthotopic PANC-1/M model (Fig. 8F). Representative tumor images also showed reduced tumor burden in the combination arms of all three models (Fig. 8G–I). Endpoint tumor weights further confirmed the enhanced antitumor effect of combination therapy in the subcutaneous PANC-1/M and MIA PaCa-2 models (Fig. 8J,K). Similarly, in the orthotopic PANC-1/M model, the combination of HuL001 at 30 mg/kg with GEM had a better therapeutic efficacy than GEM at 10 mg/kg alone, as reflected by lower tumor weights (Fig. 8L). Mechanistically, combination treatment more effectively suppressed both intratumoral lactate accumulation (Fig. 8M–O) and the levels of active TGFβ (Fig. 8P–R) compared to GEM alone in these three models. In contrast, total TGFβ levels, including active and inactive forms, were largely unchanged across treatment groups in all three models (Fig. 8S–U), indicating that HuL001 specifically interferes with TGFβ activation rather than overall TGFβ production. These findings demonstrate that HuL001 enhances the anti-tumor efficacy of gemcitabine through dual suppression of tumor metabolism and TGFβ activation. This combinatorial benefit of HuL001 supports the potential clinical value of targeting ENO1 to overcome the limitations of chemotherapy in PDAC.

## 4 Discussion

PDAC remains a treatment challenge, with a five-year survival rate below 10% and limited options. Here, we identify α-enolase (ENO1) as a clinically relevant surface protein in advanced PDAC. We show that HuL001, a first-in-class humanized monoclonal antibody, remodels the PDAC TME by targeting TAMs to disrupt tumor-supportive mechanisms. Mechanistically, HuL001 inhibits ENO1-mediated plasmin generation, inactivating downstream TGFβ, intervening metabolic reprogramming, and disrupting the pro-tumorigenic environment shaped by cancer metabolism, cytokines, and TAM-associated signaling (Fig. 7C). Notably, HuL001 blocks paracrine signaling between PDAC cells and TAMs, mitigating the TGFβ-driven vicious cycle that builds a supportive tumor “fortress.” These findings position ENO1 as a key regulator of TGFβ in PDAC progression and support HuL001’s clinical development as monotherapy or combination therapy.

To our knowledge, this is among the first studies delineating the ENO1-plasmin-TGFβ axis in remodeling the PDAC TME by integrating metabolic reprogramming with immune signals. ENO1 functions beyond glycolysis as a surface plasminogen receptor promoting plasmin generation and matrix degradation in cancers [35,36]. Likewise, plasmin-mediated activation of latent TGFβ has been documented in inflammatory and fibrotic contexts, such as alveolar macrophages during lung injury, where plasmin cleavage was essential for converting latent TGFβ to its active form [37]. Recently, Wang et al. showed Ciwujianoside E suppresses TGFβ1 activation in Burkitt lymphoma via ENO1-plasminogen disruption [38]. In PDAC, we demonstrate ENO1 mediates plasmin-dependent TGFβ activation while being TGFβ-upregulated, forming a feed-forward loop driving TME construction via immunosuppressive TGFβ signaling, M2 polarization, and metabolic reprogramming-PDAC hallmarks [39]. Thus, the ENO1-plasmin-TGFβ axis constitutes a novel and therapeutically targetable driver in PDAC. Inhibition of ENO1 reprograms the tumor microenvironment to potentiate combination chemotherapy regimens.

TGFβ activation, a tightly regulated multi-step process, drives PDAC progression via immune evasion, stromal remodeling, and metabolic reprogramming [33]. Latent TGFβ complexes with latency-associated peptide (LAP) and requires extracellular activation (e.g., integrins, reactive oxygen species (ROS), matrix metalloproteinases (MMPs), plasmin) to signal [40,41]. TGFβ-targeted therapies in PDAC, including TGFβRI inhibitor galunisertib (LY2157299) with gemcitabine [42] or durvalumab [43], and ligand-neutralizing antibody NIS793 [44], showed modest efficacy with toxicity issues. Another TGFβRI inhibitor vactosertib has shown promise in preclinical PDAC models by reducing EMT and immunosuppressive myeloid infiltration [45,46], but the challenge remains to balance therapeutic benefit with on-target toxicity. In contrast, HuL001 targets PDAC-enriched ENO1 to block plasmin-dependent latent TGFβ activation in the TME, attenuating tumor-specific signaling without systemic interference. HuL001 disrupts the TGFβ-ENO1 feed-forward loop promoting immunosuppressive macrophage polarization and glycolytic reprogramming, offering spatially confined TME modulation to overcome conventional TGFβ inhibitors’ efficacy-toxicity limitations. 

Targeting the TME is key in PDAC therapy due to its role in immune evasion and resistance [4,47]. TAMs promote progression via immunosuppression and matrix remodeling [48]. However, CSF1R and C-C motif chemokine receptor 2 (CCR2) inhibitors, which deplete or reprogram TAMs, are limited by compensatory recruitment and systemic toxicities [49]. HuL001 selectively disrupts ENO1-mediated PDAC-monocyte crosstalk, impairing TAM-associated tumor-supportive signaling without broad immune disruption. Together, these findings support the concept that targeting surface ENO1 may modulate both metabolic and monocyte/macrophage-associated components of the PDAC tumor microenvironment. Although CAFs are known to play a major role in PDAC desmoplasia and immune exclusion [50], efforts to target CAFs, such as inhibition of the hedgehog pathway or fibroblast activation protein (FAP), have yielded mixed outcomes due to CAF heterogeneity and context-dependent effects [24]. Consistent with this concept, our previous study in multiple myeloma showed that extracellular ENO1 promoted CAF-associated stromal reprogramming through the plasmin/TGFβ axis, supporting a broader role of ENO1 in tumor–stromal crosstalk [51]. The potential crosstalk between ENO1, TAMs, and CAFs warrants further investigation. While ENO1 activates TGFβ, it has TGFβ-independent roles; future work must dissect ENO1-TGFβ crosstalk in PDAC. Immune checkpoint inhibitors (ICIs) fail in PDAC due to T cell exclusion [52]. Meanwhile, ENO1 induces regulatory T cells (Tregs) and upregulates PD-L1 expression via HIF-1α [53,54,55]. Thus, HuL001 offers multifaceted TME targeting via ENO1, disrupting TAMs, stroma, and checkpoints. 

Nonetheless, this study has limitations. First, a formal statistical power calculation was not performed for the animal studies, which may limit the statistical robustness of the *in vivo* comparisons. Second, different co-culture formats and cell seeding ratios were used for distinct experimental purposes, which may have affected the magnitude of the observed responses. Third, although our data support the involvement of TAMs in PDAC, M2 macrophage immunostaining was not performed in the clinical tissue cohort, and further tissue-level validation is warranted. In addition, because protein-level validation of CD163 and CD206 was not performed in the co-culture system, our findings should be interpreted as evidence of monocyte/macrophage-associated tumor-promoting signaling rather than definitive M2 macrophage classification, although recent PDAC co-culture studies support the emergence of CD163/CD206-positive immunosuppressive macrophage phenotypes in similar contexts [56]. Fourth, although additional validation was performed in MIA PaCa-2 cells, the full set of co-culture, functional, and *in vivo* experiments was not conducted in parallel across both PDAC cell lines. Thus, the mechanistic conclusions of this study remain primarily based on PANC-1/M, with supportive findings from MIA PaCa-2. Fifth, although nude mouse xenograft models retain residual non-T-cell immune compartments, their T-cell deficiency limits comprehensive assessment of HuL001’s effects on adaptive immunity, immune checkpoints, and T-cell-mediated antitumor responses. Future work using immunocompetent or genetically engineered models is needed to evaluate its impact in a functional immune system. Sixth, the absence of patient-derived xenograft (PDX) models precludes analysis of PDAC’s histological and molecular heterogeneity; PDX studies will be required to assess ENO1 variability and responsiveness. Additionally, while HuL001 suppresses primary tumors, its effects on metastasis and recurrence remain unexplored.

## 5 Conclusion

Our study identifies ENO1 as a key driver of TGFβ activation, regulating TME reprogramming and metabolic support in PDAC. We propose inhibiting the ENO1-plasmin-TGFβ axis as a TME remodeling strategy combined with ICIs, chemotherapy, and antibody drug conjugates (ADCs). With HuL001 advancing in phase 1/2 trials for multiple myeloma, these PDAC findings support its clinical development for PDAC and other ENO1-expressing cancers.

## Data Availability

The data generated in the present study may be requested from the corresponding author.
